# Supplementary material for: Are carbon emissions trading and green financial instruments synergistic? -Comprehensive quantitative research based on content analysis
Source: PLoS One. 2024 Mar 7;19(3):e0298601. doi: 10.1371/journal.pone.0298601 (PMC10919601; doi:10.1371/journal.pone.0298601)
Supplement: S1 Data — (ZIP) [file pone.0298601.s001.zip › Data/地级市Z_F工作报告.zip!/║■─╧/┬a╡╫╩╨/└┤╫╘╣1⁄2╓┌║┼í╛┬φ┐╦╩2╛▌═°í┐_2012.docx]

各位代表：

现在，我代表市人民政府，向大会作政府工作报告，请予审查，并请各位政协委员和列席人员提出意见。

一、2011年工作回顾

2011年，是“十二五”的开局之年。面对复杂多变的宏观经济环境和兄弟市州竞相发展的态势及各种因素带来的不利影响，全市上下深入实践科学发展观，按照省委、省政府的决策部署，在市委的坚强领导下，坚定实施“科学发展、加速赶超”战略，全力推进经济社会发展和民生改善，圆满完成了市三届人大三次会议确定的各项目标任务。

（一）市域经济稳健增长

全年实现生产总值830亿元（预计数，下同），增长12.5%，超过年初目标0.5个百分点。其中第一、二、三产业增加值分别增长3.6%、16%和10.6%。

投资拉动有力。扎实开展“项目建设年”活动，全力以赴抓项目，千方百计促投资，全年建设项目1230个，新开工项目820个。争取国家和省定项目98个、资金80.58亿元。各类金融机构贷款余额478亿元，比年初新增80.5亿元。发行市政项目建设债券12亿元，实现了向资本市场直接融资的新突破。利用土地筹融资12.5亿元。累计完成固定资产投资420亿元，增长34%，超过年初目标14个百分点，为助推经济发展提供了有力支撑。同时，成功争取我市纳入衡邵干旱走廊综合治理规划、国家老工业基地规划研究范围，涟源市列入第三批国家资源枯竭城市，冷水江市继续享受国家资源枯竭城市财政转移支付政策，新化县、涟源市、冷水江市列入武陵山片区区域发展与扶贫攻坚规划，双峰县列入全省扶贫开发重点县，娄底经济开发区列入省级承接产业转移示范区，为我市经济持续发展和争取项目资金，创造了有利条件，拓展了新的空间。

消费促动趋旺。积极落实促进消费的各项政策，“家电下乡”、“家电以旧换新”销量分别居全省第1位和第2位。农贸市场标准化改造全面启动，中心城区早元、三元、中兴、育才等一批综合市场投入使用。“万村千乡市场工程”标准化农家店新增680家，“网上供销社”乡镇加盟店达125家，我市获批商务部农产品现代流通综合试点城市，城乡消费环境明显优化。全年实现社会消费品零售总额258亿元，增长17.8%，增幅居全省第4位。

开放带动加强。大力招商引资，实际到位外资1.8亿美元，到位内资150亿元，分别增长11.1%和35.9%。央企对接步伐加快，“九娄合作”顺利启动，第三届“湘博会”成功举办，与东盟、港澳、沿海的交流合作进一步加强，我市的知名度和外向度进一步提升。原产地证签证点设立。首家离岸服务外包企业入驻。全年实现进出口总额21.5亿美元，增长49%，增幅和总额分别居全省第1位和第3位。

（二）发展质量明显提升

全年完成财政总收入70.25亿元，增长24.8%；其中一般预算收入38.6亿元，增长28.6%。规模工业经济效益综合指数达到220，实现利润30亿元，创国际金融危机以来最好水平。

产业结构日趋优化。一、二、三产业结构调整为14.4:55.8:29.8，工业化率提高1.8个百分点。完成全部工业增加值420亿元，增长16%，规模工业企业达到575家，实现增加值380亿元，增长18%，工业对经济增长的贡献率达到68%；完成工业技改投资180亿元，增长30%，新增产学研创新战略联盟合作基地3个、高新技术企业7家、湖南名牌产品10个，成立首家省级工程技术中心；高新技术产业增加值占规模工业增加值比重、园区工业占工业增加值比重分别提高2个和1.5个百分点，六大传统产业占工业增加值比重、万元规模工业增加值能耗分别下降2个和7个百分点，工业结构呈现“两升两降”的新变化。全市粮食播种面积392万亩，总产157.2万吨，连续8年实现增产增收，双峰县被评为全国粮食生产先进县，新化县被评为全省粮食生产标兵县；种植蔬菜46.61万亩、经济作物90.64万亩，涟源市成为全国丰产油茶示范基地县市；湖南黑猪规模养殖场达30个，出栏黑猪10万头，湘中黑牛存栏4.4万头，新建标准化养殖小区60个，养殖业占农业总产值的比重达到59.75%；市级以上龙头企业达103家，完成农产品加工销售额73.2亿元；“三品一标”农产品质量安全认证达到89个。房地产、交通运输、邮电通信、批发零售等传统服务业平稳发展；金融、现代物流、文化创意等新兴服务业加快发展；旅游接待人次首次突破1000万，总收入57亿元，新化县进入全省旅游强县和旅游十佳县。

“两型”建设加快推进。水府示范片区建设步伐加快，出台万宝新城概念性设计与控制性详规，完成甘桂路、大井路等路基工程，仙女大道、高丰路开工建设。仙女寨悠活五星级酒店、华鑫绿能塑钢护栏、新世界建材城等项目签约进驻。水府庙国家湿地公园列入国家湿地保护补助试点单位。推进锡矿山地区和涟钢周边环境综合整治，加快资水、涟水、孙水等重点流域水污染综合治理，争取国家、省地质灾害和矿山地质环境治理专项资金1.1亿元，湘江流域重金属污染综合治理资金1.6亿元，垃圾填埋场渗漏液处理、污水污泥处理等13个项目纳入国家投资计划。削减二氧化硫3.08万吨、化学需氧量817吨、氮氧化物722吨、氨氮106吨，完成了省定任务。爱国卫生和城乡整洁行动全面铺开。积极开展“三边”造林和“四旁”绿化，造林16.6万亩，森林覆盖率达到48.15%。

安全生产持续巩固。坚持“以人为本”抓安全，出台进一步加强安全生产监管能力建设的实施意见和加快推进煤矿“三化”建设的决定，加大安全生产投入，深入开展重点行业专项整治，规范整顿矿业开发秩序，抓好安全标准化、示范化创建，全力夯实基层基础，强化企业主体责任，安全生产事故起数下降32.39%，杜绝了重特大事故，我市以及涟源市均被评为全省安全生产先进单位，实现了安全生产与经济效益的双赢。

（三）发展基础不断夯实

交通路网加快完善。完成交通建设投资87.6亿元。长昆客运专线娄底段和洛湛铁路娄邵线改造加快建设。娄新、新溆、安邵、娄长等4条高速公路全面推进。强力加快潭邵高速娄底连接线建设，实现提前通车。207国道、024县道改造完成，省道209、210、217、225、312改造全面扫尾。公路危桥改造和桥梁安保工程扎实开展。完成农村通畅工程1279.3公里。娄底机场通过中南民航局规划定点选址，涟水复航列入省规划，资水航道等级提升、大洋江航电枢纽等工程列入全省内河水运发展规划。

城镇建设扎实推进。以“三个创建”为抓手，娄底中心城区吉星、甘桂路网、孙水河南岸及防洪堤、第一污水处理厂二期、燕子岩生活垃圾填埋场等76个重点城建项目加快推进，完成投资23.3亿元。珠山公园一期竣工开园，成为深受市民欢迎的休闲健身场所和天然绿色氧吧，为城市增添了一道亮丽景观。城区新增绿地面积132万平方米，绿化覆盖率达到39.8%。创建国家园林城市顺利通过实地考查验收，创建国家卫生城市、全国文明城市工作有序推进。完成娄底市城市总体规划修改，中心城区控制性详规覆盖率达到90%。加大违法建设整治力度，进一步规范户外广告、渣土运输、交通秩序和社区物业管理，加强征地拆迁遗留问题处理，环境卫生明显好转，城市品位不断提升。县市城区建设步伐加快，冷水江市城市东扩和企业退城进郊、涟源市北扩东延南完善、双峰县五大区域板块建设、新化县打造文化旅游新城等举措卓有成效。

农村基础持续改善。扎实推进79个省级“千村示范工程”建设村和6个市级重点示范村建设，投入8700万元。统筹城乡发展、整体推进新农村建设试点在冷水江市取得突破，投入2.3亿元，首批35个先行推进村的“六个一体化”建设成效显著，成为全省统筹城乡发展的新典范。双峰县、新化县、娄星区全国小农水重点县建设全面推进，我市被列为全省水利改革试点市，完成农村水利建设投资4.1亿元。白马灌区配套与节水改造工程加快推进，治理病险水库18座，清淤扩容山塘2814口，解决20.43万人的饮用水安全问题。村级活动场所、村卫生室建设覆盖面不断扩大，广播电视“村村通”加快推进。森林防火、农林病虫害防治、动物疫病防控、气象水文信息服务、农业技术推广、农机具监理、农资供应价格监管等工作进一步加强。农民负担监管、“三冬”生产等工作扎实开展。我市集体林权制度主体改革获全省一等奖。

要素保障切实加强。强化用地保障，共呈报各类建设用地项目152个，总用地1533公顷，批回125个，总用地932.5公顷，确保了重点工程项目的用地需求。实施土地综合整治与开发项目73个，争取专项资金1.6亿元，新增耕地649公顷，连续12年实现耕地总量占补动态平衡。加快园区基础建设，完善园区产业配套，完成投资90亿元，新建标准厂房20万平方米，为企业入驻和承接产业提供了有效平台。天然气入娄管输工程开工建设。电网建设得到加强，通信网络不断完善，煤、电、油、运、气等要素保障有力，全力以赴调煤保电，全年完成680万吨电煤调配任务。

（四）社会事业全面进步。基础教育巩固发展，建设农村义务教育合格学校71所，维修改造农村中小学校舍124所。千方百计解决市县（市）城区“大班额”问题，新增学位4500个，中心城区城南中学、吉星路小学建设前期工作全面启动，县市优化整合教学资源工作取得实效。职成教育加快发展，成功组建娄底职业教育集团。高等教育稳步发展，湖南人文科技学院获批硕士点。科技事业加速发展，我市被纳入国家农村农业信息化示范工程建设示范区域，通过“全国科技进步市”考核，获省科技奖励3项，其中获得省技术发明奖一等奖1项，实现该奖项零的突破。双峰县、新化县被评为全国科技进步先进县，冷水江市被列为全国科技富民强县。深入推进医药卫生体制改革，实现基本药物制度全覆盖，基层医疗机构全面推行基本药物零差率销售，公共卫生、农村卫生、社区卫生均衡发展，卫生监督、妇幼保健、基础免疫工作扎实有效。文化事业再上新台阶。大型花鼓戏《花落花开》参加建党90周年湖南优秀剧目展演，梅山傩戏入选第三批国家级非物质文化遗产保护名录，新增20处省级文物保护单位。新建42个乡镇综合文化站，建成605家农家书屋，成功举办第三届曾国藩文化旅游节。广电传媒、新闻出版、书画艺术蓬勃发展，影响力进一步提升。省第十二届运动会筹备工作全面启动，市体育中心正式投入运行，成为展示娄底城市形象的新地标。人口计生综合改革不断深化，整体水平稳步提高，全国青少年健康人格工程试点工作成效明显，市计生协会宣传工作被评为全国先进。食品药品监管不断加强，药品安全专项整治获全国先进。关心下一代、老龄、老区、教育扶助、红十字会、慈善、地方志等工作取得新成效，市老促会及双峰、涟源等老促会被评为全国先进，娄星区获全国方志系统先进集体。坚持用群众工作统揽信访工作，领导干部接访深入推进，信访积案化解经验全国推广，非正常上访综合考核位列全省第一，信访工作继续保持全省先进。扎实推进社会治安综合治理，严厉打击各类违法犯罪，加强社会治安防控体系建设，遏制了重大恶性案件发生，治安状况进一步好转，应急管理稳步推进，社会大局和谐稳定，双峰县获全省社会治安综合治理先进县并保持平安县称号。

（五）民生改善卓有成效。圆满完成政府绩效评估与为民办实事各项任务。财政对农业、社保、医疗、教育、住房保障等民生支出达到87.23亿元，占一般预算支出的69.32%。千方百计稳定和扩大就业，新增城镇就业人员3.45万，帮助失业人员实现再就业1.89万，城镇零就业家庭保持动态清零，新增农村劳动力转移就业5.45万。城镇基本养老、基本医疗、失业、工伤、生育保险制度进一步完善。城市、农村低保月人均补差分别比去年提高38元、37.2元。城镇居民养老保险和新型农村社会保险试点积极推进，新农保参保人数120.43万。新型农村合作医疗参合率达到98.2%，受益人数200万，统筹地区政策范围内平均住院费用报销比例达到71.87%。社会救助进一步加强，救助流浪人员1.9万。新建和改扩建乡镇敬老院12所，完成农村危房改造7100户，开工建设保障性安居房48728套，竣工28293套。提取住房公积金3.1亿元，发放贷款6.7亿元，支持4800户家庭解决住房困难。财政投入扶贫资金4700万元，争取移民后扶资金2.3亿元，受益移民32万人。关心困难群体生活，启动了低收入群体价格补贴联动机制。农民人均纯收入达到4000元，城镇居民人均可支配收入达到16900元，分别增长20%和12.5%。

（六）自身建设不断加强。主动接受人大法律监督、工作监督和政协的民主监督，认真办结市三届人大三次会议议案2件、人大代表建议216件、市政协常委建议案1件、政协委员提案147件。积极支持各民主党派、工商联、无党派人士参政议政。高度重视社情民意，认真倾听群众呼声，着力解决了中心城区三中三小地下人行通道等群众关心、关注的问题。大力推进依法治市，全面启动“六五”普法，加快转变政府职能，进一步规范权力运行，在全省率先出台法治政府建设若干规定，率先建立行政复议、行政诉讼、信访工作联席会议制度，率先实施军转干部安置改革，坚持行政事业单位凡进必考，市本级全面实行刚性收费、刚性预算，法治政府建设取得新成效，得到省委、省政府的充分肯定。行政复议工作获全国先进。加强政务公开和电子政务建设，市信访与政务中心竣工。深入开展“创先争优”和“效能制度落实年”活动，集中整治“三强”行为，发展环境不断优化。严格执行党风廉政建设责任制，政府廉政建设持续加强。加大经济责任审计、行政监察力度，完成政府投资工程结算审计项目390个，核减资金2.49亿元，完成财务收支、专项资金、经济责任等审计项目226个。

国防动员和后备力量建设扎实推进，党管武装工作创新发展，履行使命能力持续提升，公安、消防、武警队伍建设不断加强，为全市经济社会发展做出了重大贡献。国资监管、统计、民族宗教、人防、对台、外事侨务、档案、保密、残联、地震、仲裁和工会、共青团、妇女、儿童等各项工作均取得了新的进步。

各位代表，过去一年成绩的取得，是市委坚强领导和市人大、市政协有力监督支持的结果，是全市广大干部群众心血和汗水的结晶，也离不开社会各界人士的关心和支持。在此，我代表市人民政府，向全体人大代表、政协委员和全市人民，向各民主党派、工商联、无党派人士，向驻娄部队指战员、武警、消防官兵、公安干警，向中央、省属单位的各位朋友，向所有关心、支持娄底建设和发展的同志们、朋友们，致以崇高的敬意和衷心的感谢！

成绩有目共睹，但问题也不容忽视，主要是：经济总量还不大，综合实力还不强；产业结构还不优，发展方式仍然粗放；要素“瓶颈”制约明显，发展基础依然薄弱，个别项目进展比较缓慢；可用财力比较紧张，民生改善任务很重；影响和谐稳定的因素依然突出，社会管理亟待加强；政府职能转变还不够，干部作风还需进一步改进，发展环境还需进一步优化，等等。对此，我们务必高度重视，努力加以解决。

二、2012年目标要求

2012年是实施“十二五”规划承上启下的重要一年。综观国际国内宏观环境，我们既面临严峻的发展挑战，更面临难得的历史机遇。当前，世界经济形势总体上十分严峻复杂，世界经济复苏的不稳定性不确定性上升，国内稳增长、调结构、控通胀的压力仍然很大。但我们也应看到，中央在保持政策的连续性和稳定性的同时，进一步增强了调控的针对性、灵活性、前瞻性，明确了稳增长、控物价、调结构、惠民生、抓改革、促和谐的总体方针和稳中求进的主基调，再次强调扩内需、促消费的工作重点，这为我市对接国家政策来争取项目和资金支持，带来了难得的历史机遇。只要我们保持清醒头脑，加强风险评估，切实抢抓机遇，聚精会神搞建设，一心一意谋发展，竭尽全力惠民生，就一定能够赢得发展的新优势、开创发展的新局面、实现发展的新跨越。

2012年政府工作总体要求是：坚持以邓小平理论和“三个代表”重要思想为指导，深入贯彻落实科学发展观，认真贯彻中央决策部署和省第十次党代会、市第四次党代会及中央、省委、市委经济工作会议精神，把“稳中求好、好中求快”作为总基调，全面推进“四化两型”建设，加快对接融入长株潭，着力保增长、提质量、增效益，着力转方式、优结构、上水平，着力扩投资、强基础、增后劲，着力惠民生、促和谐、谋幸福，进一步深化改革开放，切实加强和创新社会管理，实现全市经济社会发展速度、质量、效益、水平的新突破，在“科学发展、加速赶超”、建设幸福娄底的伟大征程上，迈出更加坚实的一步。

2012年的主要预期目标是：生产总值增长12.5%以上；规模工业增加值增长18%以上；全社会固定资产投资增长25%以上；财政一般预算收入增长13.5%以上；社会消费品零售总额增长18%以上；进出口总额增长15%以上；城镇居民人均可支配收入、农民人均纯收入分别增长11.5%、13%以上；人口自然增长率控制在7‰以内；节能减排完成省定目标。

实现以上目标任务，在工作中要牢牢把握以下原则：

第一，坚持“两型”引领，转变发展方式。把资源节约、环境友好作为衡量经济发展的第一准则，紧紧围绕科学发展主题，全力推进经济发展方式转变，以水府示范片区建设引领全市“两型”社会建设，打造“三基地一中心一枢纽”。

第二，坚持工业主导，壮大产业支撑。把做大做强新型工业作为促进我市“科学发展、加速赶超”的第一引擎，以信息化促进新型工业化，以新型工业化带动农业现代化、新型城镇化，加快园区建设，努力构建现代产业体系。

第三，坚持投资带动，增强发展后劲。把上项目扩投资作为保增长、促转型的第一抓手，做实项目前期，狠抓项目争取，加快项目建设，以项目扩投资、调结构、强基础、增后劲，努力推进全市经济社会持续快速健康发展。

第四，坚持改革开放，激发发展活力。把深化改革扩大开放作为提升市域经济影响力的第一选择，全面树立开放包容意识，充分调动一切积极因素，为发展提供更加灵活、更加有效的外部支持和体制机制保障。

第五，坚持协调发展，建设幸福娄底。把以人为本、建设幸福娄底作为政府工作的第一目标，更加注重社会和谐与民生改善，更加注重生态建设与城乡统筹，更加注重解决实际问题，维护群众利益，努力提高人民生活质量。

三、2012年工作重点

围绕目标要求，今年要着力在五个方面实现新突破。

（一）在扩大有效投资、夯实发展基础上取得新突破。要竭尽全力破解资金瓶颈，以超常规举措扩投资、抓项目、保增长，确保全年完成固定资产投资525亿元以上，力争达到550亿元。

千方百计拓宽筹资渠道。积极抢抓政策机遇，全力以赴向上争取资金，提高争取项目的针对性和实效性，力争国家和省对我市的投入在去年的基础上有新的增加。切实加强银企合作，推动金融机构加大有效信贷投入，确保全年各项贷款增长20%以上。重点支持招商银行、华融湘江银行和交通银行等在娄设立分支机构，力争每个县市都设立一家村镇银行，完成娄底、双峰农村商业银行的筹建工作。抓好湖南省金融生态良好城市试点，力争涟源市和双峰县成功创建省级金融安全区。大力提升资本运作能力，充分运用政策性银行信贷融资、担保融资、项目融资、出让土地使用权融资、特许经营权融资等多种融资方式，努力扩大融资规模。发挥市城投集团的平台作用，确保全年筹融资12亿元以上，力争突破15亿元。积极扶持有潜质的企业上市融资，力争年内实现我市企业上市零的突破。着力创新方式方法，千方百计对外引资，更加注重投资强度和单位土地面积投资对财政的贡献率，重点引进世界500强、央企、优质上市公司和大型民营企业，确保实际到位外资与内联引资分别增长30%和40%以上。鼓励、激活和规范民间投资进入基础产业、基础设施、市政公用事业等领域。加强财源建设，强化税收征管，确保财政收入稳定增长，严格执行财政预算，调整优化支出结构，进一步发挥财政资金对扩大投资的引导作用。

把准把实投资重点。集中推进列入全市“三个一”行动计划的重大项目。着力加强以交通为重点的基础设施建设。确保娄新高速公路在国庆节前竣工通车；加快推进长昆客运专线娄底段、洛湛铁路娄邵线改造、新溆、安邵、娄长高速公路建设；下大力争取娄衡、娄益高速公路实质性开工建设；力争涟源湄江至新化琅塘高速公路、涟源荷塘至娄新高速娄底西互通至丰瑞干线公路、新化资江三桥、冷水江资江二桥等项目开工建设；全面启动省道210锁石至界首上等“十二五”国省干道改造，做好安张衡铁路、洛湛铁路娄益线改造、引资济涟调水工程、白马水库扩容提型、资水河道疏浚、涟水复航、安化至武冈高速公路新化段等项目的前期工作；加快天然气入娄管输工程建设，开工建设娄底至双峰、涟源至冷水江至新化天然气管道项目。着力加强以新型工业为重点的产业项目建设。确保华润雪花啤酒、中兴液压扩建、文昌科技扩建、华南煤机与方瑞钢管扩建、湖南煤机综采设备生产线、国藩水工机械等项目建成投产；加快推进涟钢汽车板电工钢、红太阳新材料产业园、三一中源新材料产业园二期、金华特种车辆、巨大重工机械、大丰和绿色动力科技园、闪星锑业有色系列产品、天宏不锈钢、回春堂药业扩能改造、高盛板业、农友机械扩建、乙撑胺生产线、龙腾机械制造产业园、和盛菌业产业园等项目建设；扎实做好薄板深加工产业园、涟源国产实业二期扩建、冷水江电子科技产业园、金电四号机组，以及大熊山、龙山、新化温塘与冷水江风电场等项目的前期工作。加强以民生保障为重点的社会事业建设。加快推进全市保障性住房建设、娄底市污泥无害化处理与综合利用、冷水江与双峰人民医院异地扩建、长青社区服务中心等项目；启动涟钢周边失地农民再就业基地、恩口煤矿特殊困难群体产业基地、中心城区城南医院、新化中医院搬迁等项目；切实做好娄底城市垃圾焚烧发电沼气发电及综合处理、双江水库直饮水工程、新化县城饮用水取水口上移、两丝水库工程等项目的前期工作。通过大力实施一批重点项目，既扩大有效投资，又切实改善发展的基础条件。

全力推进项目建设。强化抓项目就是抓投资、抓项目就是抓发展的理念，完善项目前期工作协调机制，落实好“一个项目、一名领导、一套班子、一抓到底”的工作制度，加强项目调度，加快工作进度，加大服务力度，让投资尽快形成实物工作量。落实《娄底市国有土地上房屋征收与补偿实施办法》和《娄底市集体土地上房屋拆迁补偿安置办法》，坚持“安置先行、同步推进”，加强失地农民的社会保障，有力化解征迁矛盾，确保项目早落地、早开工、早竣工、早见效。坚持节约集约用地，加强土地储备，盘活存量土地，优先保证工业用地。狠抓项目监管，确保工程质量。加强煤、电、油、运、气等生产要素的调控和调度，不折不扣完成省定调煤保电任务，坚决杜绝因要素保障不及时而影响项目建设进度。

（二）在优化产业结构、实现转型发展上取得新突破。要全力以赴加快产业升级换代，构建现代产业体系，提高新型工业占第二产业、现代农业占第一产业、第三产业占整个经济的比重，走出一条转型发展的新路子。

全力推进新型工业。加快促进传统优势产业改造升级，大力实施“153211”工程，加快推进企业技术改造，重点抓好涟钢、冷钢技改，双峰海螺、湖南海螺二期、华新水泥异地扩建，金电机组脱硫改造、涟源区域煤矿瓦斯发电，以及杨家山、大跃井、斗笠山煤业开发等煤电一体化工程，力争完成工业技改投资增长25%以上。加快农机、煤机、特种陶瓷、耐火材料等行业兼并重组，积极组建专业产业园基地。落实信贷、税收等各项扶持政策，支持中小微型企业加快发展，确保新增规模工业企业60家以上。大力发展战略性新兴产业，把培育和壮大战略性新兴产业作为产业结构优化升级的重要突破口，落实关于培育战略性新兴产业发展的意见，用好战略性新兴产业财政专项引导资金，大力发展先进装备制造、新材料、电子信息、节能环保、生物医药等产业，着力培育一批战略性新兴产业示范企业，力争全年战略性新兴产业占生产总值比重达到8%以上。充分发挥企业主体作用，巩固科技创新战略联盟成果，加强与央企、高校、科研院所的技术创新合作，加大高新技术产业培育和产品开发，争取高新技术企业达到45家以上、高新技术产业增加值占规模工业增加值比重达到30%以上。扎实开展“园区建设年”活动，积极落实市委、市政府进一步推进产业园区发展的若干意见，完善体制机制，抓好调规、扩区和基础设施建设，在提升产业承载力和集聚力上实现新突破，确保全市园区建设标准厂房50万平方米以上，完成工业项目投资100亿元以上，园区规模工业增加值增长30%以上，固定资产投资增长40%以上。

加快发展现代农业。重点做好“大、特、新、深”四篇文章，在产业化、规模化、品牌化上取得新突破，力争农业增加值增长4%以上。加强耕地保护，遏制耕地抛荒，鼓励土地依法自愿有序流转，培育种养殖大户，加快基层农技推广体系建设，支持专业基地建设，推广先进节能环保农业机械，力争全年粮食播种面积410万亩、总产量168万吨以上，新增蔬菜种植面积5万亩以上，生猪、肉牛出栏分别增长3%、5%，丰产油茶、南竹、工业原料林等三个100万亩基地效益不断提高；扎实推进农田水利建设，加快白马、梅山等大型灌区，九峰、星星、红卫、光辉、余元等72座小型水库，段江等大型灌溉排水泵站及田间配套工程建设。大力推进结构调整和品种改良，突出抓好“两黑”产业，出栏黑猪15万头以上，存栏湘中黑牛5万头以上；抓好中药材、黑米、黑茶、珍稀食用菌、葛根、软籽石榴等特色农产品生产，着力打造富有地域特色的产品品牌，力争新增省级以上农产品知名品牌2个以上；启动中国航天科技集团太空育种研究中心和航天生态农业种子示范基地建设。促进传统农业与服务业等产业相融合，以乡村旅游富民工程为龙头，大力发展观光农业、休闲农业和生态农业，力争营业收入增长20%以上。大力发展农产品精深加工，力争新增市级以上农业产业化龙头企业10家，农产品精深加工产业集群产值增长30%以上，加快打造长株潭重要的农产品供应基地；加快发展农民专业合作组织，鼓励“公司+基地+农户”的发展模式，抓好农产品现代流通综合试点，加快娄底中心城区湘中果蔬批发大市场、涟源蓝田农贸市场、新化城南市场、冷江市场、双峰永丰市场等31个农贸市场标准化改造，继续实施“农超对接”、“新网工程”和“万村千乡市场工程”，加快“网上供销社”、乡镇直营店和社区便利店建设，构建农产品流通便捷通道，切实解决农资供应“买难”和农产品外销“卖难”问题。

全面提升第三产业。立足区位优势和交通基础，坚持抓特色、树品牌、上档次，力求在打造区域性物流与商贸集散中心建设上取得新突破，力争第三产业增加值增长11.5%以上。大力发展现代物流，加强物流设施、物流配送及物流信息平台建设，重点抓好湘中国际、万宝消费品、娄底鑫业、双峰丰厚、涟源机电、新化卷烟中转站等物流园和湘中国际汽贸城的开工建设，确保天客物流建成运营。培育本土商贸服务品牌，引进大型商贸流通企业，进一步扩大零售、批发业辐射半径，着力激活城乡消费。加快发展金融、法律、会计、中介等生产性服务业，改造提升餐饮、住宿等传统服务业，大力发展电子商务、连锁经营、家政服务、信息、咨询、创意等新兴服务业。规范房地产市场的管理，促进房地产业健康发展。大力发展旅游业，推进旅游与文化融合发展，打造娄底旅游知名品牌，把文化旅游业培育成市域经济的支柱产业。注重引进战略投资者参与旅游资源开发与保护，规划启动水府国家湿地公园建设，抓好曾国藩故里、梅山龙宫、紫鹊界、湄江、波月洞、大熊山、龙山、仙女寨、大乘山、雷峰山等景区的综合开发，启动紫鹊界申报AAAA景区工作，积极构建大梅山文化旅游协作区，加强市域旅游的整体规划、策划、包装和市场营销，加强重点旅游线路和景区景点配套设施建设，力争接待游客突破1200万人次，实现旅游收入65亿元以上。

着力推进信息化。力争完成“数字娄底”建设总体规划和相关专项规划，大力促进信息化与工业化的融合，运用信息技术改造提升钢铁、有色、建材、煤炭等传统产业，提高装备水平和技术实力。以统一城市通讯管道资源建设为突破口，抓好“五平台一中心”等信息基础设施建设。大力培育和引进电子信息龙头企业，加快打造电子信息材料、电子元器件等电子信息产品制造业集群。

（三）在强化“两型”统筹、促进协调可持续发展上取得新突破。要切实加快“两型”建设，以“两型”引领新型城镇化和新农村建设，统筹城乡发展和区域发展，加强生态文明建设，强化资源的循环利用，提高资源环境承载能力，促进市域经济协调可持续发展。

更大力度推进“两型”示范区建设。完成万宝新区和东部新区起步区控制性详规编制，全面启动8平方公里起步区基础设施建设，加快仙女大道、高丰路、甘桂路和薄板产业园、绿色动力科技园园内道路的建设与完善，推进投资主体多元化、资金来源多渠道、投资方式多样化，着力在资源节约、环境友好、产业优化、科技创新和土地管理等重点领域和关键环节先行先试、率先突破，加快打造生态优良、产业配套、功能完备、舒适怡人的山水生态新区。大力发展“两型”产业，落实“两型”产业发展规划，优化项目和企业布局，严格资源承载力和环境容量限制，严格执行环境准入和“三同时”制度，加快创建“两型”产业技术研发和推广应用公共服务平台，力争在“两型”产业发展上取得新进展。

更大力度推进新型城镇化。切实强化规划引领。加强规划的刚性控制，加快形成以总体规划为核心、近期建设规划为重点、片区控制性详规为基础的城乡规划体系，抓好专项规划的完善配套，确保控规全覆盖，以规划引领城镇化有序发展。切实加强基础建设。按照扩容提质的要求，加快中心城区“南扩北延、东优西联”步伐，完成投资28亿元以上，力争金子塘至湘中大道、吉星路北段涟水河大桥、娄涟公路开发区段改造等城市内环线项目建成通车；大力实施中心城区街道综合改造提质美化“三年行动计划”，强力推进城中村改造和拆迁安置遗留问题处理，按照统一规划、逐步实施的原则，启动1－2个城中村改造工程，提升城市品位，进一步增强中心城区对市域经济发展的带动力。加快县市城镇路网、防洪、垃圾、污水处理等基础设施建设，重点推进冷水江东扩新区路网、涟源城区沿河风光带、双峰县城第二水源、新化城东防洪堤及沿江公路等项目，进一步完善城镇功能，抓好规划建设管理示范镇创建工作，支持促进劳动密集型产业、农产品加工业向中心镇和重点镇集聚，增强县市城区和小城镇的带动力与辐射力。切实加强城镇管理。按照城镇管理精细化、标准化、规范化的要求，继续深化城管综合执法，积极推进市政维护、园林绿化、垃圾处理、环卫清扫等公用事业市场化运作，加强城区交通整治，着力打造“生态宜居，安全适业”的区域性中心城市。

更大力度推进新农村建设。以实施“百城千镇万村”新农村建设工程试点为契机，以冷水江市统筹城乡发展、整体推进新农村建设试点为示范，加大试点乡镇、村（片）建设力度，抓紧规划编制，加快新农村建设有序推进。抓好农村道路改造、改水改厕、房屋风貌改造、植树造林等工作，强力推进农村环境卫生专项整治，逐步推进规模化畜禽养殖场和养殖小区的工程减排，扎实推进乡村清洁工程。加强农村建房规划引导和控制，规范村民适度集中建房。全面加强农村公共设施建设，建立健全农村社会化服务体系，加快城市公共设施和公共服务向农村延伸。加强扶贫开发和移民后扶工作，全面执行新的扶贫标准，严格落实移民后扶政策，以特困区、库区、移民区、山区为重点，加大政府投入、信贷投放和财政贴息规模，进一步整合各类扶贫资金和项目，全力构建专项扶贫、行业扶贫和社会扶贫“三位一体”的大扶贫格局，力争减少贫困人口3.5万。

更大力度加快县域经济发展。着力推进“一心两翼”城镇带建设步伐，加快涟源、双峰与娄底中心城区的“融城计划”，启动娄涟公路改造和省道312线升国道工作，推动双峰县城至长昆客运专线娄底南站城际公路建设的前期工作，着力打造“娄－涟－冷－新”和“娄－双”两条经济发展黄金走廊，带动县域经济的腾飞。支持各县市区发挥资源优势和比较优势，大力发展县域特色产业，加快园区建设，走专业化园区之路，突出政策向园区倾斜、企业向园区集中、产业向园区集聚，进一步提高园区对县域经济的贡献率。积极落实扩权强县改革政策，扩大县域发展自主权，加大对县域经济发展的政策扶持力度，优化县域发展环境，增强县域经济发展活力。

更大力度强化生态环境保护和能源资源节约。以解决饮水安全问题和空气、土壤污染等影响群众健康的突出环境问题为重点，加大生态环境综合治理力度。加快推进湘江流域重金属污染、锡矿山地区砷碱渣污染、双峰县原坳头山磺矿历史遗留污染和资水、涟水、孙水等流域的综合治理。下决心保护好白马、大江口、双江、车田江、周头湖等供水水源，解决农村安全饮水25万人。统筹推进城乡绿化，人工造林16万亩，退耕还林1.85万亩，高标准完成水府庙生态与植被恢复，加强森林防火，确保森林覆盖率稳定在48.15%以上、森林活立木蓄积量增加30万立方米，达到1123万立方米。深入推进节能减排，严格责任目标考核，坚决落实“一票否决”。大力发展绿色经济、低碳经济和循环经济，积极申报国家循环经济试点城市和国家再生资源回收体系建设试点城市。推进娄星区循环经济工业园、涟源市汇源循环经济工业园、冷水江市循环经济产业园建设。鼓励支持建筑节能和机关节能，争创国家可再生能源建筑应用示范城市。

（四）在注重改革开放、激发发展活力上取得新突破。要在更大空间和更深层次上加快改革，扩大开放，最大限度激发社会创新、创造、创业活力，切实提高发展的竞争力和外向度。

进一步解放思想。思想解放的程度，决定工作的力度、发展的速度。要进一步加强学习，组织关键部门、关键岗位的同志到沿海发达地区挂职，学习先进理念，改进工作方法，缩小我市与发达地区在思想观念、工作理念上的差距。要进一步更新观念，敢于打破思想禁锢，破解各种制约瓶颈，让不断迸发的先进思想汇聚成推动科学发展的大潮。要进一步开阔视野，努力克服封闭保守、小富即安的心理，跳出娄底看娄底，以更大的勇气来加速赶超，以最大的魄力来推进发展。

扎实推进各项改革。启动财政预算绩效管理改革试点，稳步推进财政信息公开。强化国有企业和行政事业单位国有资产管理体制改革，加强国有资产监管，防止国有资产和收益流失，并实现保值增值。积极推进省属下划企业和市属国企改制扫尾工作。稳步推进资源性产品和公用事业价格改革，运用价格杠杆，促进经济发展方式转变。继续深化人事制度改革，推行事业单位岗位设置管理和新进人员公开招聘制度，完善以考试考核相结合的军转干部安置方式。推进文化行政管理体制和文化市场综合执法改革，深化公益性文化事业单位内部劳动人事、收入分配和社会保障制度改革，稳妥推进经营性文化单位转企改制。深入推进医药卫生体制改革，全面实施国家基本药物制度，加强基层医疗卫生服务体系建设，进一步完善新型农村合作医疗制度。全面推进水资源管理、水利投融资、水利工程建设管理、基层水利服务体系管理、水价等体制机制改革。逐步完善乡村公路管养体制。

大力提高开放水平。积极搭建交流平台，争取将“湘博会”升格为省级经贸展会。更加注重招商选资，突出产业链、生态环保、先进技术等领域招商。继续抓好与中粮、五矿、有色金属研究总院、中国供销集团等大型央企项目合作，着力引进战略投资者，提高利用外来资金的质量和水平。大力实施“走出去”战略，继续拓展深化与东盟的经贸合作，加强与港澳台、长三角、珠三角等地的区域合作，鼓励和支持有实力的企业走出去。加快发展加工贸易与服务外包等外向型经济，积极承接沿海产业转移，进一步加大双峰台资工业园等承接产业转移园区的建设和引资力度。加强农机、煤机、特种陶瓷、薄板深加工、锑产品深加工和制鞋业等六大出口基地建设。加快娄底铁路口岸建设，早日实现“属地报关、口岸验放”的大通关运行模式，实现投资贸易便利化，努力构建开放型经济发展的新格局。

切实优化发展环境。着力优化市场环境，积极倡导诚实守信的良好风气，依法整顿和规范市场经济秩序，坚决打击制售假冒伪劣商品、偷税骗税、欺行霸市等违法犯罪行为。全力优化建设环境，严厉整治涉企“六乱”和“三强”行为，坚决制止和拆除违法建筑，严厉查处损害经济发展环境典型案件，为企业生产和项目建设保驾护航。着力优化人才环境，落实人才规划纲要，以推进十大人才工程为抓手，引进、盘活和用好各类人才。着力优化创业环境，鼓励、支持、引导非公经济发展，倡导和尊重全民创业，抓好创业基地建设，力争新增国家创业基地1－2家，省级创业基地3－5家。落实支持创业的各项扶持政策，为一切创业者提供最宽松的环境、最优惠的政策、最便利的条件。

（五）在推进民生改善、增进人民福祉上取得新突破。建设幸福娄底是全市人民的共同期盼。要想民之所想，忧民之所忧，不空谈，实作为，为人民群众多干实事，干好实事，努力提高全市人民的幸福指数。

全面推进社会事业发展。继续推进建设教育强市工作，高度重视和突出解决娄底中心城区、各县市城区的读书难和“大班额”问题，实施中心城区化解“大班额”四年行动计划，今年启动建设项目21个，加快城南中学、吉星路小学建设进程，新增学位8705个；优化农村教学资源，加快农村合格学校建设和中小学危房改造步伐，着力改善农村办学条件。落实学前教育三年行动计划，构建学前教育公共服务体系；推进娄底技师学院建设，加强职业教育基础能力建设，促进特殊教育、民办教育、高等教育发展；加强学校周边环境和校车安全专项整治，为学生提供一个安全、舒适的学习环境。加快推进文化大发展大繁荣，全面提升我市文化软实力。启动市本级“三馆一中心”等文化工程和县市区“二馆一中心”及文化站建设，配送农家书屋977家，实行文化场馆免费开放服务，继续抓好广播电视“村村通”和数字电视建设工程，提高公共文化服务能力。保护、开发和利用好本土文化资源，办好“蚩尤故里、天下梅山”和曾国藩文化旅游节，充分发挥文化领军人物的作用，深度挖掘民间文化艺术资源，努力创作更多的文化精品，着力打造地域特色文化品牌。鼓励和支持文化企业发展，做大做强文化产业。完善公共卫生服务体系，加强基本公共卫生服务，加强重大疾病防控，提高卫生应急处置能力，确保不发生甲类传染病和大面积传染病暴发流行。继续实施“降消”项目，进一步降低孕产妇和婴儿死亡率，落实好农村孕产妇免费住院分娩政策。注重基层医院和城区现有医院服务能力的提升。着力推进“三个创建”，巩固创园成果，攻坚克难创建国家卫生城市，为创建全国文明城市夯实基础。扎实做好第十二届省运会各项筹备工作，加强竞技队伍建设，提高竞技水平，抓紧推进县市区体育场馆及配套工程建设。

努力提高民生保障水平。切实为民办好实事，严格对照省里下达的任务要求，抓好责任分解，加强资金配套，强化督促检查，确保民生项目全面落实。加快建立劳动力和用工信息统一平台，扩大劳动就业，加强技能培训，确保全年新增城镇就业3万人，新增农村劳动力转移就业5万人，城镇零就业家庭实现动态清零，城镇登记失业率控制在4.5%以内。完善社会保障体系，在全市范围内实施新农保和城镇居民养老保险，实现养老保险全覆盖，扩大医疗生育保险覆盖面，在全市范围内开展居民医保门诊统筹。加大社会救助力度，积极推进城乡低保扩面提标。大力发展慈善事业。进一步加快残疾人综合服务体系和社会保障体系建设步伐，继续抓好白内障免费复明手术和0－6岁残疾儿童抢救康复项目。全面启动事业单位和民间非营利组织参加工伤保险，扩大征缴覆盖面。将失业保险扩面的重点转移到非公有制企业、改制企业、事业单位，建立失业动态监控体系和失业调控预警机制。进一步扩大住房公积金覆盖面，支持更多城镇职工解决住房困难。构建和谐劳动关系，加大劳动监察执法力度，推进劳动人事争议仲裁机构实体化建设，切实保障进城务工人员的合法权益。加大“米袋子”、“菜篮子”工程建设力度，全力实施“菜篮子”工程三年行动计划。继续加大物价调控力度，完善粮油、肉类、蔬菜等生活必需品的供给和储备制度，居民消费价格涨幅控制在5%以内。强化食品药品监管，加快建立食品药品城乡一体化监管网络，确保食品药品安全。加快农村危房改造和保障性住房建设，新建公租房6000套、廉租房3454套，城市棚户区改造10200户，国有工矿棚户区改造3995户，国有林场棚户区改造683户，新增廉租房货币补贴1800户，让更多低收入群众住有所居。

着力促进农民增收。不折不扣落实粮食直补、良种补贴、农机补贴等各项强农支农惠农政策，确保农民政策性增收。鼓励、支持、引导大学生、城镇居民和返乡农民到农村创业，以创业促增收。大力发展劳动密集型产业、农村商贸物流等服务业和小型微型企业，增加农民就近就业机会。普遍提高农民素质，有针对性地加强农民技能培训，提高农民就业能力，增加广大农民的务工收入。通过拓宽农民增收渠道，努力实现农民收入增加与经济增长同步。

扎实抓好生产安全。坚持把安全发展作为科学发展的基本要求，严格落实安全生产责任制，强化安全监管和属地管理，加大安全生产投入，加强安监队伍建设，提升安全生产总体保障能力。继续推进娄底安全发展示范市和企业安全生产标准化创建工作，全面开展安全生产金牌、银牌企业评比和授牌活动，继续深化矿山、道路与水上交通、烟花爆竹、危险化学品、特种设备、消防、建筑施工等重点行业领域安全专项整治。扎实推进城市工业灾害防治，深化煤矿瓦斯灾害治理和“三化”建设，加强农村交通安全管理和其他重大危险源的监控治理，消除安全隐患，严防重特大事故发生。

切实加强社会管理。紧紧围绕三年内建成全省社会管理先进城市的目标，加快完善、落实和创新各项社会管理措施，抓好娄星区综合试点。继续推进信访工作体制机制创新，启用“一大厅三中心”，进一步落实领导干部定期接访、信访积案化解制度。加强社会治安综合治理，筑牢治安防控体系，强化乡（镇）、村和街道社区治安管理，夯实基层基础，把矛盾纠纷消除在萌芽，解决在基层。加强应急管理与应急体系建设，提高预防和处置突发事件的能力。继续深入开展打黑除恶专项行动，提高人民群众的安全感。加强社区建设，全面推进小区物业管理。加强人口计生工作，切实稳定低生育水平，扎实抓好人口计生综合改革、青少年健康人格工程、整治“两非”、计生优质服务和幸福家庭建设等重点工作。进一步完善全员人口数据库，不断加强对流动人口的管理与服务，切实保障人口安全。持续开展国防教育，切实做好“双拥”工作，着力抓好国防动员和民兵预备役工作，推进人防工程建设，促进军民融合式发展。做好老干部服务和关心下一代工作，加强未成年人保护。重视革命老区工作。加强统计工作，充分发挥统计在经济社会发展中的基础作用。统筹抓好民族宗教、对台、外事侨务、地方志、档案、保密、气象、水文、仲裁、防震减灾和红十字会等工作。

四、加强政府自身建设

新形势、新任务给政府工作提出了新的更高要求，要以更高标准、更实措施、更严要求加强自身建设，全面提高政府的行政效率和施政水平，不断开创政府工作的新局面。

更加注重依法行政，建设法治政府。认真执行人大及其常委会的决议和决定，自觉接受人大及其常委会的法律监督、工作监督和政协的民主监督，认真办理人大代表议案、建议和政协委员提案。全面落实依法行政实施纲要，深入开展“六五”普法工作，严格按照法定权限和程序履行职责、行使权力，讲法律、讲规矩、讲效率。严格执行决策听证、公众参与、专家咨询、集体决策制度，坚持科学民主决策。继续强化刚性收费、刚性预算，加快出台招投标、招拍挂、城市规划、征地拆迁等方面的刚性规定，推进公共资源交易平台建设，确保行政权力运行公开透明、依法规范。广泛听取各民主党派、工商联、无党派人士意见，支持工会、共青团、妇联等人民团体发挥桥梁纽带作用。以法治促和谐，切实维护人民群众合法权益。

更加注重工作落实，提高行政效能。加快转变政府职能，把工作重心向促转型保增长倾斜、向重服务保民生倾斜，真正谋大事、办实事、解难题、促发展。大力弘扬务实求效的工作作风，顺应民意，敢于担当，说了就干，干就快干，干就干好。改进会风文风，少开会、少发文，少搞检查评比，集中精力保重点，握紧拳头抓大事。坚决强化行政问责，强化督查督办，强化日常监督，确保重大决策落实到位、群众合理诉求解决到位、投诉事项处理到位，坚决杜绝“有令不行、有禁不止”和“肠梗阻”现象，确保政令畅通。把人民的满意度作为检验标准，问政于民、问需于民、问计于民，摸实情，出实招，办实事，求实效，切实解决老百姓最关心、最直接、最现实的问题。大力开展政府工作绩效评估，推动机关效能建设的规范化、经常化和制度化，加快电子政务服务和电子监察系统建设，确保政务中心高效运行，落实限时办结、首问责任、部门协作、服务承诺、绩效考核等制度，完善“一厅式审批、一站式服务、一票制收费、一体化运行”模式，努力让群众只进一张门、办好一切事。

更加注重廉洁从政，维护政府形象。严格落实党风廉政建设责任制，完善教育、制度、监督并重的惩治和预防腐败体系，进一步严肃廉政纪律，严格执行廉政准则，坚定不移开展反腐败斗争。加强对行政审批、工程招标、土地出让、产权交易、政府采购、资金监管等重点领域和关键环节的廉政监察、审计监督和财政监管，遏制“潜规则”、破除“关系网”，让腐败行为无处可行。大兴勤俭节约、艰苦朴素之风，坚决反对铺张浪费、大手大脚，不搞劳民伤财的形象工程，不搞贪大求洋的面子工程，开源节流保重点，集中财力干大事，把有限资金和资源用到促发展、强基础、惠民生上来，着力打造人民满意政府。

各位代表！过去的成绩铸就了今天坚实的起点，未来的美好图景需要我们用智慧和汗水去精心描绘。市第四次党代会已全面吹响加快富民强市、建设幸福娄底的前进号角，让我们在市委的坚强领导下，以敢打硬仗的勇气，以狠抓落实的作风，以力求实效的韧劲，凝心聚力，开拓创新，奋发进取，开创又好又快发展的新局面，为早日实现幸福娄底的宏伟目标而努力奋斗！
